# Efficacy and Safety of a New Crosslinked Hyaluronic Acid Dermal Filler for Facial Volume Restoration: A Prospective Clinical Trial

**DOI:** 10.1093/asjof/ojag082

**Published:** 2026-05-07

**Authors:** Ander Pino, Josune Torrecilla, José María Alonso, Linda Tovito, Amaia Huguet, Raúl Pérez

## Abstract

**Background:**

Hyaluronic acid is commonly used as a dermal filler for aesthetic purposes. Crosslinking allows a better performance once injected into the skin. A new filler, known as BtHCROSS, has been developed using a novel crosslinking technology (SARE technology).

**Objectives:**

To clinically assess the efficacy and safety of BtHCROSS for facial volumization.

**Methods:**

Two hundred and three volunteers with signs of facial aging received a single injection of BtHCROSS. Follow-up visits were established at 21 days, 6 months, and 9 months. Efficacy assessment included the Mid-Face Volume Loss Scale (MFVLS), Wrinkle Severity Rating Scale (WSRS), and Lip Fullness Grading Scale (LFGS). Global Aesthetic Improvement Scale (GAIS) and Likert’s patient satisfaction questionnaires were also registered. Biometric parameters were assessed using Visioscan, Cutometer, Moisturemap, and Visioface tools. Adverse events related to the study treatment were registered throughout the duration of the study.

**Results:**

Mid-Face Volume Loss Scale and WSRS scores showed a statistically significant improvement at both 6 and 9 months. Early noticeable results were also observed after 21 days in LFGS and WSRS scales. The specialist MD reported a GAIS improvement in more than 88%, 92%, and 76% of the volunteers at 21 days, 6, and 9 months, respectively, and the satisfaction percentage was above 70% in all cases. Biometric analysis showed a rapid and statistically significant improvement, maintained during the follow-up period.

**Conclusions:**

This study demonstrates that a single injection of BtHCROSS is safe and effective for the management of facial volumization for up to 9 months.

**Level of Evidence: 2 (Therapeutic):**

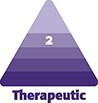

In recent years, a youthful appearance has become a major concern for general population, which presents a challenge for aesthetic clinicians. During the aging process, shifting of connective tissue components such as collagen, elastin, and elastic fibers or glycosaminoglycans takes place, and facial volume loss contributes to the formation of folds, wrinkles, and expression lines that become increasingly prominent.^1^ In addition, the extracellular matrix of the dermis undergoes natural alterations, including changes in dermal fibroblast metabolism and, which altogether leads to a decrease of natural hydration. This decline results in the reduction of dermo/epidermal thickness, which in turn causes adipose tissue sliding and resorption over facial areas such as cheek, jaw, and chin.^2^ Additionally, repeated muscle activity, along with changes in bone musculature, subcutaneous tissue and skin, leads to volume loss in the mimetic facial areas, such as the perioral/periorbital area, lips, nasolabial folds, frown and marionette lines. In addition, the face contains many muscle groups affected by gravity, making it a challenging region to maintain volume and firmness.^3^

Currently, there are multiple skin rejuvenation techniques. Dermal fillers are the most widespread and effective option for the management of facial volume restoration and soft tissue augmentation. Injectable fillers are minimally invasive, require little to no downtime, and pose minimal risk, providing a temporary and nonsurgical option that does not require incisions nor general anesthesia.^4^ This allows individuals to return to their activities within a few hours after the procedure, making fillers a good alternative to surgical interventions. In this sense, hyaluronic acid (HA) is a natural glycosaminoglycan that is used in numerous dermal fillers,^5^ with growing popularity and an increasing number of products available on the market. In recent years, global demand for HA volumization has significantly risen, becoming almost a fundamental practice for any aesthetic physician. In this regard, HA-based devices have become synonymous with effectiveness and safety, ranking among the top nonsurgical interventions in the field of aesthetic medicine.^6^

An optimal dermal filler must fulfill certain attributes. It needs to be biocompatible, nonpermanent but long-lasting, adaptable to the facial anatomy, with reduced side effects, easy to apply and cost-effective in a single injection basis.^7^ Although there are numerous commercially available HA-based fillers, it is not easy to fulfill all these characteristics in a single product. Consequently, they are commonly injected in a repeated basis or used synergistically in combination with other procedures such as facelifts, laser resurfacing, or peelings.^8^ As a logic result, clinical studies are still being performed with the aim of optimizing the rheological and biomechanical profile of HA fillers to achieve a better clinical performance when used as monotherapy and on a one-shot basis.^9^

Several studies have accordingly demonstrated that the chemical modifications of HA chains result in a consistent matrix that allows a better performance in terms of volume restoration and soft tissue augmentation once injected into the skin.^10^ In contrast to linear polymers commonly used for skin boosting, HA can be covalently and physically crosslinked, providing improved stiffness and viscoelastic properties to prolong the volumetric effect of the hydrogel. There are different and coexistent approaches that nowadays focus on this biochemical stabilization. In this context, a novel crosslinking technology has recently arisen, known as SARE technology.^11,12^ This technology has been preclinically assessed and optimized to develop a new crosslinked dermal filler known as BtHCROSS.

In the Basque language, “SARE” means “network”, which adequately resembles the inner structure of BtHCROSS. Recent studies have evaluated the physicochemical and rheological performance of this product vs other commercially available crosslinking technologies and dermal fillers such as Juvederm Volume (Allergan Aesthetics, Irvine, CA), Belotero Soft (Merz Aesthetics, Raleigh, NC), Restylane Refyne (Galderma, Zug, Switzerland), or Teosyal Ultimate (Teoxane, Geneva, Switzerland).^13^ The results concluded that BtHCROSS presents an engineered viscoelasticity in terms of elastic and loss modulus that offers a predictable and adaptative clinical performance. In addition, it has a balanced durability and optimal enzymatic resistance, resulting from the enhanced modification degree of its polymer chain, thereby ensuring reliable results and a precise control during administration.^13^ Hence, once injected intradermally, this 3-dimensional matrix aims at providing a natural, adaptable, stable and long-lasting volumetric support that counteracts the facial deflation, laxity and cutaneous atrophy derived from the aging process.

The goal of this study is to clinically assess the efficacy and safety of a single application of BtHCROSS in the management of facial volume restoration.

## METHODS

### Study Design

This was a postmarket and prospective clinical trial. It was carried out in the Complutense Medical Center (Virtus Group, Madrid, Spain). This trial was reported following the Strengthening the Reporting of Observational Studies in Epidemiology (STROBE) Statement.^14^ The study protocol (code BtHCROSS-PIC01-2021) as well as the subject information sheet and informed consent form, were reviewed and approved on March 30, 2022 by the ethics committee of the University Hospital Principe de Asturias (code PIPS03/2022, Madrid, Spain) in accordance with the international ethical standards from the revised World Medical Association Declaration of Helsinki amended in 2013. This study was conducted following the guidelines established in UNE-EN ISO 14155:2021 (Clinical research on medical devices for humans. Good clinical practices) and CPMP/ICH-GCP-E6/135/95 (Guidance on Good Clinical Practice of the European Medicines Agency).

### Subject Selection

Informed consent (including photographs) was obtained prior to study entry. At baseline, the participants underwent an initial individual assessment, and the medical history was documented. Selection criteria for study entry are depicted in Table 1. After the treatment visit, subject's follow-up visits were scheduled at 21 days, 6 months, and 9 months. Blinded researchers that were not involved in the application of treatments conducted the data processing of the clinical results.

**Table 1.** Inclusion and Exclusion Criteria for Study Participation

Inclusion criteria	Exclusion criteria
Men or women between 30 and 70 years of age. Subjects that present facial aging symptoms including decreased radiance, hydration, or firmness. Willingness and ability to complete questionnaires and understand study instructions. Signed informed consent specific to the study.	Pregnant or breastfeeding women. Allergy or sensitivity to hyaluronic acid or any other ingredient of the product under study. Have undergone any rejuvenation or wrinkle correction procedure in the 6 months prior to the study entry or during the study such as radiofrequency, electrotherapy, botulinum toxin, tensor threads, platelet-rich plasma, or laser techniques. Autoimmune or inflammatory diseases that comprises the condition of the skin. Disease or active infection in the area to be treated. Lymphatic and/or vascular pathology. Previous facial surgery. Blood coagulation disorders or in treatment with anticoagulants. Disproportionate expectations regarding the clinical performance of the product.

### Interventions

BtHCROSS is a CE marked Class III medical device that is based on a crosslinked hyaluronic acid dermal filler that is obtained using the SARE technology. Depending on the final sodium hyaluronate concentration, BtHCROSS is presented in 3 models of 1 mL prefilled syringes: BtHCROSS1.5% (15 mg/mL), BtHCROSS1.75% (17.5 mg/mL), or BtHCROSS 2% (20 mg/mL). BtHCROSS is used in a one-shot basis with the possible requirement of an additional retouch session depending on the patient requirements and professional criteria.

All subjects who were included were scheduled for the baseline visit and received the treatment reported in this study, consisting in a single injection of BtHCROSS, with an optional corrective retouch after 3 weeks depending on case-by-case requirements. Following the manufacturer’s instructions for use, each product was applied to a different anatomical region to which its formulation is more appropriate; BtHCROSS 1.5% was applied in lips and perioral/periocular area, BtHCROSS 1.75% was administered to nasolabial folds, marionette lines and frown, and BtHCROSS 2% was used for deep volume restoration in cheeks/cheekbones, chin, and jaw. As each concentration is intentionally designed and applied for distinct anatomical regions and assessed with different aesthetic scales and clinical endpoints, no statistical comparison between concentrations or sites was performed. Furthermore, the study was not designed to compare the different concentrations against each other, but rather to evaluate their performance within their intended indications, thus no site- or concentration-based differences have been outlined that could influence outcomes. Nonetheless, such comparisons could be of interest for future research where an inclusion of a control group would strengthen both site and concentration dependent variables from a statistical point of view. The injection technique can be described as an intradermal or subdermal deposition of the product, flowing the appropriate procedure depending on each anatomical treatment area. An example of such techniques is scraping, which uses appropriate needles in a linear retrograde mode. After infiltration, the product was evenly distributed by slight pressure application, to achieve the desired clinical efficacy.

### Outcome Measures

#### Efficacy Assessment

Assessments were performed in a controlled environment with standardized and controlled humidity and temperature. Efficacy outcomes were assessed at baseline and during the follow-up visits at 21 days, 6 months, and 9 months. The primary efficacy outcome was the clinical improvement regarding specific and validated aesthetic scales fulfilled by clinicians. The 5-point Mid-Face Volume Loss Scale (MFVLS), Wrinkle Severity Rating Scale (WSRS), and Lip Fullness Grading Scale (LFGS) were used for the clinical assessment of BtHCROSS 2%, 1.75% and 1.5%, respectively.

In addition, the Global Aesthetic Improvement Scale (GAIS) was fulfilled by the clinicians (percentage reporting slight improvement, improvement or important improvement was calculated as responders). A Likert’s patient satisfaction questionnaire was also completed by the volunteers (patient percentage stating slightly satisfied, satisfied or very satisfied was calculated as responders).

To quantify the aesthetic improvement, additional biometric outcomes were studied using validated aesthetic instrumental (Courage Khazaka Electronics, Cologne, Germany) such as Visioscan,^15^ Cutometer,^16^ Moisturemap,^17^ and Visioface.^18^

#### Safety Assessment

At each visit, the onset, severity, duration, and nature of adverse events was registered. To evaluate the safety profile of the treatments, all complications and adverse events were recorded with an accountability scale.

#### Statistical Analysis

Considering baseline demographics and variables, a descriptive analysis of the sample was performed. The mean, SD, and range were determined for quantitative variables, and a frequencies analysis was conducted in case of qualitative variables. Random events were considered for consecutive measurements for each volunteer. To assess treatment efficacy over time, linear mixed-event models (LMMs) were fitted. For repeated means, a 2-tailed *P*-value was calculated. A descriptive analysis was performed for clinical outcomes. Normality, homogeneity of variances and homoscedasticity analysis was performed. Depending on conditions, parametric test (ANOVA) or a nonparametric analysis (Kruskal–Wallis) was performed. The significance level was set at 0.05 and CIs were calculated at 95%. For the statistical analysis, the R-Studio version 4.3.2 and SPSS statistical package version 29.0 was used.

## RESULTS

Table 2 shows demographics at baseline. A total of 203 patients were included in the study and completed the follow-up. The mean age was 51.74 ± 9.26 years, and the female/male ratio was 90.11%/9.89%. A total of 67, 69, and 67 subjects were treated with BtHCROSS 1.5%, BtHCROSS 1.75%, and BtHCROSS 2%, respectively. The injected mean volume of BtHCROSS in each area was 1.32 ± 0.18 mL, and an optional corrective retouch was carried out in 44.3% of volunteers.

**Table 2.** Baseline Demographics of Subjects Included in the Study

Treated area	BtHCROSS 1.5%		BtHCROSS 1.75%			BtHCROSS 2%	
	Lips	Perioral/periocular	Nasolabial folds	Marionette lines	Frown lines	Cheeks/cheekbones	Chin/jaw
Sample size (N)	24	43	31	19	19	43	24
Age (years)	49.91 ± 9.67	51.42 ± 8.77	49.59 ± 10.60	58.53 ± 7.18	48.45 ± 9.03	50.71 ± 9.95	53.55 ± 9.61
Gender (%): female/male	100/0	90.7/9.3	100/0	84.2/15.8	84.2/15.8	88.4/11.6	83.3/16.7
Habits (%): smoking/sports	16.7/37.5	20.9/37.2	12.9/41.9	10.5/47.4	10.5/47.4	16.3/51.2	16.7/37.5

Table 3 summarizes safety data. During the study, no adverse events were observed apart from slight swelling, transient pain, hematoma, and itching. These are expected events considered mild to moderate and were mostly resolved within a few days following the injection. Only 2 volunteers required the administration of hyaluronidase in response to excessive swelling. These 2 events were considered by the specialist as usual after the use of dermal fillers. Vascular occlusion is a described but not very common event, mainly related to insufficient anatomical knowledge, and represents a risk for dermal fillers. Nevertheless, it is worth pointing out that no adverse events related to vascular occlusion were reported in this study.

**Table 3.** Adverse Events

	BtHCROSS 1.5%	BtHCROSS 1.75%	BtHCROSS 2%
Pain: event % treatment visit/21 days/6 months/9 months	46%/16%/0%/0%	35%/7%/0%/0%	31%/2%/0%/0%
Hematoma: event % treatment visit/21 days/6 months/9 months	21%/13%/0%/0%	1%/7%/0%/0%	12%/4%/0%/0%
Itching: event % treatment visit/21 days/6 months/9 months	10%/3%/0%/0%	13%/7%/0%/0%	6%/7%/0%/0%
Bleeding: event % treatment visit/21 days/6 months/9 months	6%/0%/0%/0%	1%/0%/0%/0%	0%/0%/0%/0%
Swelling/edema: event % treatment visit/21 days/6 months/9 months	1%/0%/0%/0%	0%/0%/0%/0%	0%/0%/0%/0%

**Figure 1.**
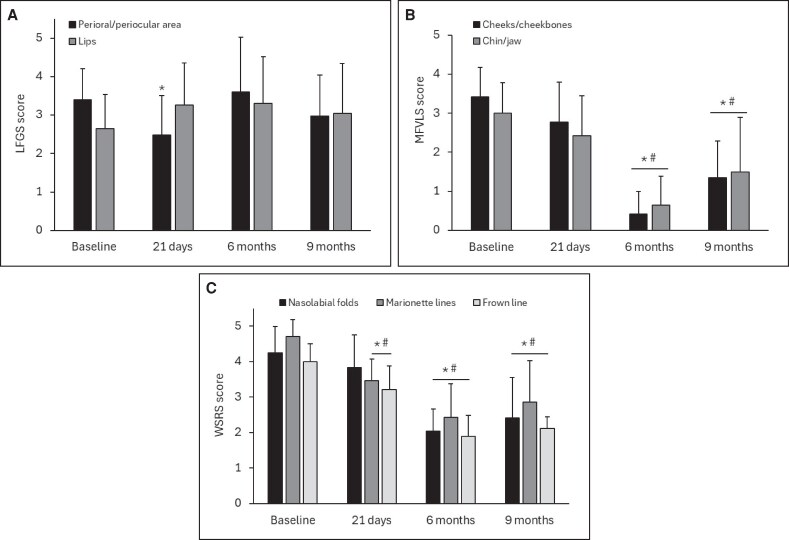
Clinical improvement for BtHCROSS treated areas assessed by specific and validated aesthetic scales. (A) Lip Fullness Grading Scale (LFGS). (B) Mid-Face Volume Loss Scale (MFVLS). (C) Wrinkle Severity Rating Scale (WSRS). An increase in LFGS score is associated to a clinical improvement. A decrease in MFVLS and WSRS score is associated to a clinical improvement. *Statistically significant differences compared with baseline (*P* ≤ .05). # ≥ 1 point improvement in the specific scale score.

The clinical improvement was assessed by specific and validated aesthetic scales fulfilled by clinicians (Figure 1). In general, a statistically significant improvement could be observed throughout the study when compared with baseline (*P* ≤ .05). Specifically, the MFVLS and WSRS scores showed a significant improvement at both 6 months and 9 months. Early results were also observable after 21 days in LFGS and WSRS scales. The most clinically relevant improvement was observed among nasolabial fold, marionette and frown line amelioration, as well as volumization of cheeks/cheekbones and chin/jaw.

**Figure 2.**
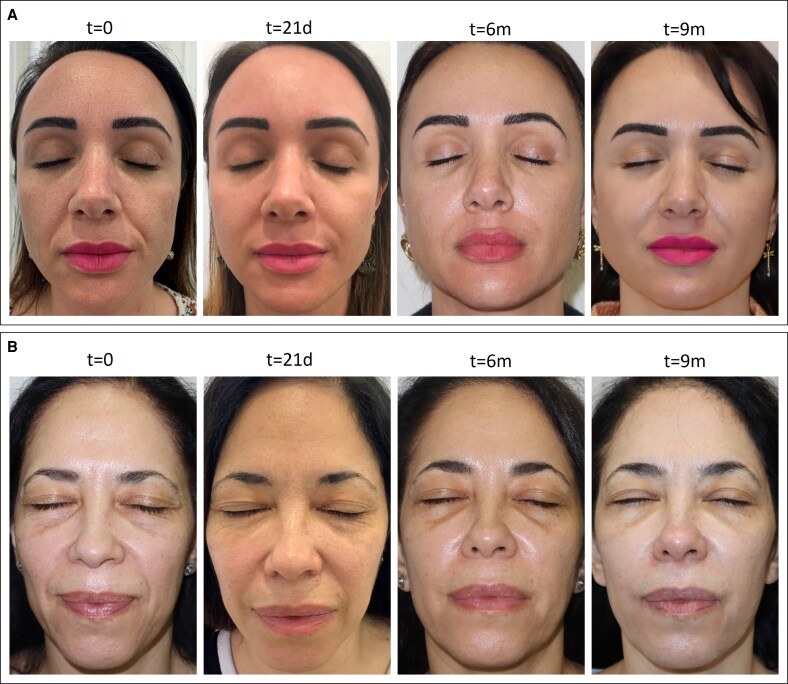
Standardized photographs illustrating the clinical evolution of 2 volunteers at different time points. (A) BtHCROSS was administered into the periocular area, nasolabial folds, frown line, cheekbone, and jaw (female, 38 years). (B) BtHCROSS was administered into perioral/periocular area and nasolabial folds (female, 53 years).

**Figure 3.**
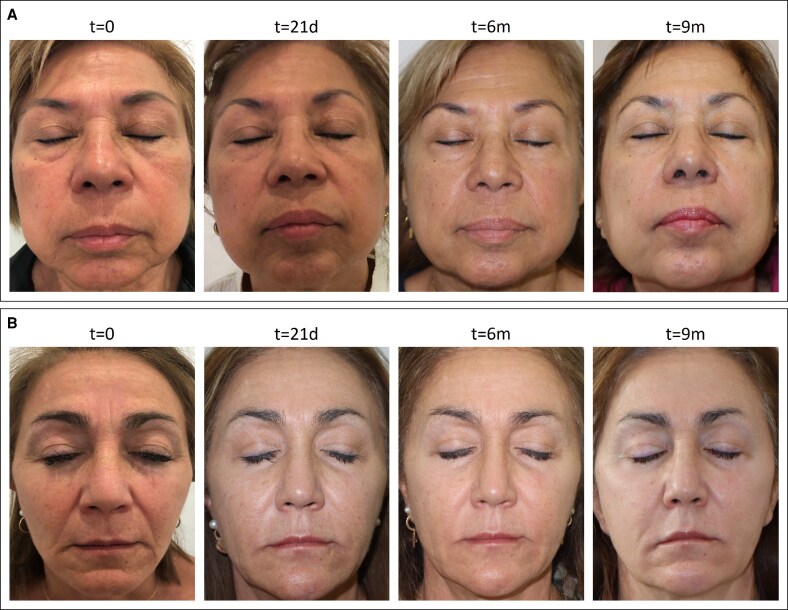
Standardized photographs illustrating the clinical evolution of 2 volunteers at different time points. (A) BtHCROSS was administered into the perioral area and marionette lines (female, 65 years). (B) BtHCROSS was administered into the lips, perioral/periocular area, nasolabial folds, marionette lines, frown line, cheekbone, chin, and jaw (female, 58 years).

When analyzing the pretreatment and posttreatment status, specialists reported a global aesthetic improvement in more than 88%, 92%, and 76% of the volunteers at 21 days, 6 months, and 9 months, respectively (GAIS scale). These results are in accordance with the subjective satisfaction referred by participants in which a responder percentage above 70% was achieved for all treated areas throughout the study (Likert’s questionnaire). Figures 2 and 3 include a set of photographs that illustrate the clinical evolution of volunteers.

**Figure 4.**
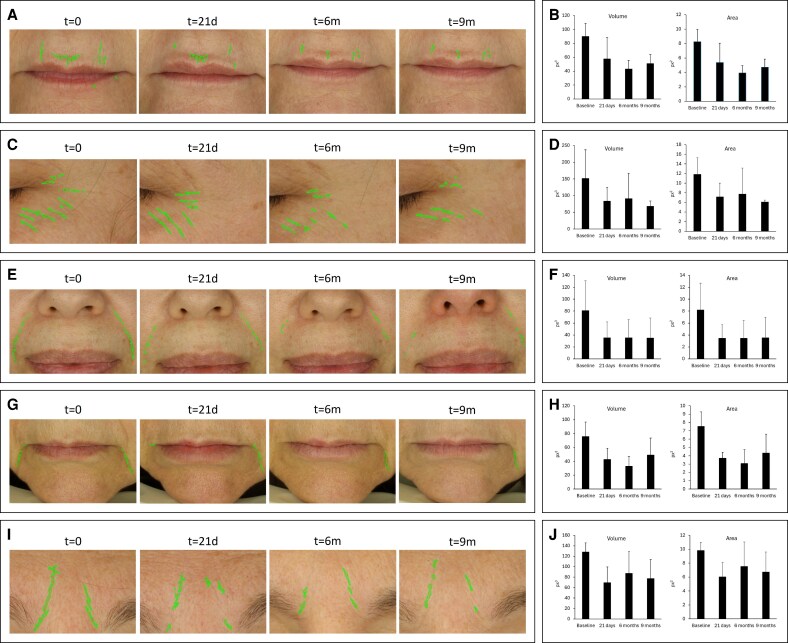
Representative wrinkle assessment using the Visioface camera. (A, B) Perioral area (female, 50 years). (C, D) Periocular area (female, 49 years). (E, F) Nasolabial folds (female, 53 years). (G, H) Marionette lines (female, 57 years). (I, J) Frown lines (female, 60 years).

**Figure 5.**
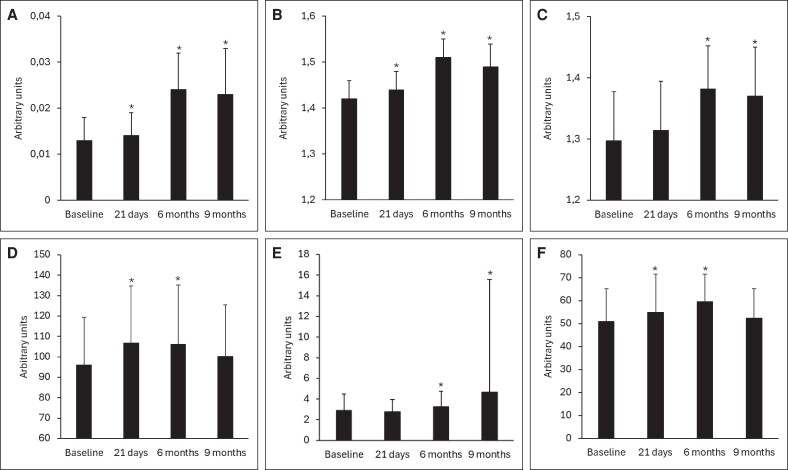
Global biometric analysis of the skin in response to BtHCROSS throughout the study period. (A) Skin rejuvenation. (B) Cutaneous uniformity. (C) Cutaneous homogeneity. (D) Water content. (E) Roughness. (F) Viscoelasticity. An increase in the biometric parameters is associated to a clinical improvement. *Statistically significant differences compared with baseline (*P* ≤ .05).

**Figure 6.**
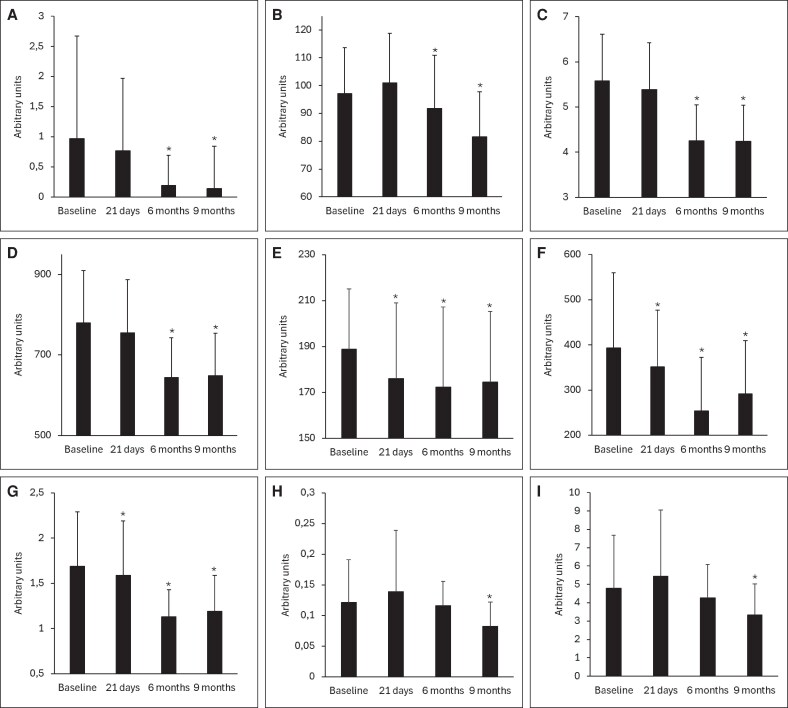
Global biometric analysis of the skin in response to BtHCROSS throughout the study period. (A) Scaling. (B) Wrinkle depth. (C) Rugosity. (D) Surface irregularities. (E) Skin hydration. (F) Skin smoothness. (G) Cutaneous condition. (H) Flexibility. (I) Firmness. A decrease in the biometric parameters is associated to a clinical improvement. *Statistically significant differences compared to baseline (*P* ≤ .05).

**Figure 7.**
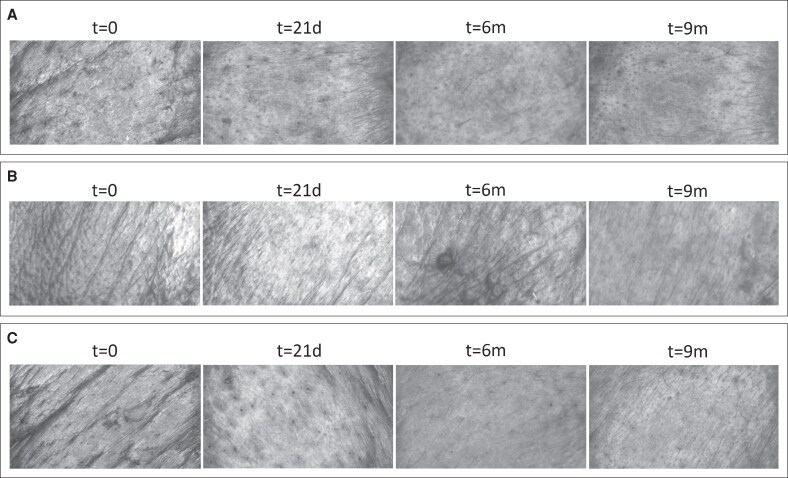
Representative gray scale images taken by biometric tools for quantitative assessment. (A) Perioral area. (B) Periocular area. (C) Marionette lines.

The biometric analysis revealed that BtHCROSS promoted a statistically significant and rapid improvement, which was maintained during the study (*P* ≤ .05). Wrinkle assessment using the Visioface camera is depicted in Figure 4. Specifically, the cutaneous uniformity, roughness, rejuvenation, and homogeneity showed a steady improvement that was maintained up to 9 months (Figure 5). The viscoelasticity and water content of the skin surface also revealed a rapid improvement at 21 days persisting for 6 months. Accordingly, the hydration index, cutaneous condition, and skin smoothness also displayed an immediate improvement, which was maintained during the entire study (Figure 6). Other outcomes such as scaling, wrinkle depth, cutaneous rugosity, or superficial irregularities also revealed statistically significant results at 6 and 9 months. This progressive improvement was additionally detected when analyzing other outcomes, such as the flexibility and firmness of the skin, which revealed significant changes by the end of the study. Figure 7 illustrates gray scale images for quantitative assessment.

## DISCUSSION

The skin suffers from the influence of genetic factors and exposure to hostile extrinsic environment such as solar radiation, pollutants, or nutrition. The cutaneous aging process is usually accompanied by structural, functional, and phenotypic changes, which derive into volume loss, wrinkles, laxity, and dermal atrophy.^2^ Hyaluronic acid based dermal fillers are widespread aesthetic treatments aimed at promoting skin rejuvenation, volume restoration, and wrinkle and folds filling. However, fillers usually face a harsh biomechanical microenvironment once injected into the target tissue that need to be addressed for an optimal clinical performance.^8^ Here, a new crosslinked dermal filler, with optimal rheological and viscoelastic properties, was assessed in 203 volunteers and for a follow-up period of 9 months.

In this study, BtHCROSS showed an optimal performance at the short (21 days), mid (6 months), and long term (9 months). In concrete, BtHCROSS promoted soft tissue restoration and cutaneous atrophy amelioration, which reduced the appearance of marionette lines, frown lines, and deep nasolabial folds. Moreover, it triggered a reduction in lid to cheek junction, thus favoring a youthful appearance. Significative chin/jaw augmentation was achieved, which helped to balance the profile of the face. Additionally, the lost volume in the midface was replaced, which helped both, creating a lifting effect and supporting the subcutaneous tissue. BtHCROSS not only revolumized the mid/lower facial surface but also achieved an overall lip augmentation and improved appearance over perioral and periocular areas. These results were evaluated by means of specific aesthetic scale scoring including LFGS, MFVLS, and WSRS and are in accordance with other studies regarding dermal filler-based deep volume restoration.^6^

BtHCROSS 1.5% was applied in lips and perioral/periocular area. The anatomy of these areas is characterized by highly vascularized, dynamic tissue with a relatively superficial plane. Therefore, intradermal techniques with retrograde linear threading or microbolus deposition were preferred, as they allow precise volume control, reduce the risk of visible irregularities, and preserve natural mobility. BtHCROSS 1.75% was administered to nasolabial folds, marionette lines and frown. In these areas, deeper planes were commonly selected. Linear retrograde or fanning techniques enabled uniform distribution of the product along the fold, minimizing the risk of overcorrection while optimizing mechanical support. BtHCROSS 2% was used in cheeks/cheekbones, chin, and jaw. In these areas, treatment aims to enhance contour and definition, requiring greater structural support. For this reason, subdermal injections with controlled bolus or deep linear deposition techniques were frequently employed. Herein, the reported data are consistent with the GAIS score achieved at the end of the follow-up. BtHCROSS improved baseline dull appearance and uneven texture leading to a volume gain that lightened the flat medial cheek, wrinkles, and deep folds. Furthermore, the sagging appearance was reduced, a fact that positively influenced the facial balance, musculature support, and deep defect replacement. In addition, this performance was not associated with remarkable adverse events. When occurred, they were almost related to the injection procedure and disappeared within a few days, as it is usually reported after the use of dermal fillers.^4^ This could explain the results obtained in patient satisfaction surveys.

Besides, several biometric analyses were carried out with specialized topographic equipment.^19^ These technologies are designed for the objective and quantitative analysis of cutaneous quality. They provide valuable data about the clinical performance of dermal fillers regarding surface softening, tone recovery, volume correction as well as information about structural/physiological changes, connective tissue status, and lifting capabilities. Concerning this, many biometric parameters revealed a statistically significant improvement in response to BtHCROSS throughout the study period not only at the short term (21 days) but also at the mid and long term (6 months and 9 months, respectively). This is the case of parameters such as skin rejuvenation, cutaneous uniformity, skin hydration, smoothness, and cutaneous condition. Other parameters such as cutaneous homogeneity, roughness, viscoelasticity, scaling, wrinkle depth, rugosity, surface irregularities, flexibility, and firmness presented a statistical improvement in the mid or the long term.

To sum up, results presented herein demonstrate that facial volume restoration with crosslinked hyaluronic acid is safe and effective, which is in accordance with aesthetic guidelines and previously reported studies.^5^ Moreover, this study suggests that BtHCROSS, which is obtained from high purity nonanimal HA by using the SARE technology, is an optimally crosslinked hydrogel that offers sustained aesthetic results. In fact, these results are obtained after a single injection of the product, thus highlighting its cost-effectiveness as a nonpermanent but long-lasting dermal filler. In this regard, previous studies have reported that the equilibrium between the chemical modification degree of polymer chains and HA concentration is essential to achieve the desired clinical efficacy.^10,20^ Moreover, the nonanimal origin, high purity, and the optimal HA ratio are also key aspects of BtHCROSS. These features, together with the swelling control and the reduced enzymatic degradation of the product, would explain the results presented herein, as the hydrogel performance depends on its stability once injected into the tissue.^12^ Furthermore, the biomimetic rheological profile in terms of viscosity and elasticity provided by the SARE technology offers a versatile hydrogel which adapts adequately to the facial anatomy, thus exerting its function in a more efficient manner. This fact, along with the physiological microenvironment that is maintained during the controlled and homogeneous stabilization process of the product, could also be related with the ease of injection and minimal side effects reported in the study. Finally, the strict quality controls that undergoes the product ensures undetectable levels of endotoxins or unreacted crosslinker, thus complying with biocompatibility standards and requirements.^11^

This study has several limitations worth mentioning, including the single-center design, lack of blinding, absence of control group, a more nuanced interpretation of the results and the absence of a subanalysis based on the distribution of different ethnic groups. Specifically, the lack of a control group precludes causal inference that limits claims of efficacy relative to existing fillers. In addition, a deeper comparison between HA concentration results and treated areas could have provided interesting insights regarding the clinical performance of each model (1.5%, 1.75%, and 2%). At this point, future studies might corroborate the potential of BtHCROSS as an effective hydrogel for tissue augmentation in comparison to other commercially available dermal fillers.

## CONCLUSIONS

This study demonstrates that a single injection of BtHCROSS is safe and effective for the management of facial volumization for up to 9 months.
